# Deciphering Cellular Heterogeneity and Communication Patterns in Porcine Antral Follicles by Single-Cell RNA Sequencing

**DOI:** 10.3390/ani13193019

**Published:** 2023-09-26

**Authors:** Na Chen, Yong Zhang, Yuhan Tian, Shumei Wu, Fei Gao, Xiaolong Yuan

**Affiliations:** 1 Shenzhen Branch, Guangdong Laboratory for Lingnan Modern Agriculture, Genome Analysis Laboratory of the Ministry of Agriculture, Agricultural Genomics Institute at Shenzhen, Chinese Academy of Agricultural Sciences, Shenzhen 518000, China; 2 Guangdong Laboratory of Lingnan Modern Agriculture, National Engineering Research Center for Breeding Swine Industry, State Key Laboratory of Livestock and Poultry Breeding, Guangdong Provincial Key Laboratory of Agro-Animal Genomics and Molecular Breeding, College of Animal Science, South China Agricultural University, Guangzhou 510000, China; 3 Comparative Pediatrics and Nutrition, Department of Veterinary and Animal Sciences, Faculty of Health and Medical Sciences, University of Copenhagen, 1870 Frederiksberg, Denmark

**Keywords:** antral follicle, granulosa cells, scRNA-seq, cell heterogeneity, cell-cell communication

## Abstract

**Simple Summary:**

Oocytes are the foundation of life in mammals. They develop in small sacs called follicles in the ovaries. Each follicle holds one oocyte and numerous accompanying cells, and the final stage of oocyte development happens in a follicle called the antral follicle. The successful growth and maturation of oocytes depend on complex interactions with neighboring cells, mainly granulosa cells. Understanding the differences among these granulosa cells within the antral follicle and how they communicate with each other is essential for comprehending how mammals reproduce and grow. In this study, we collected cells from these follicles in pigs and utilized single-cell RNA sequencing to profile the transcriptomic features of these cells. The study discovered diverse subpopulations of granulosa cells within antral follicles, and they were found to communicate extensively. These findings provide us with valuable insights into how follicles develop and oocytes mature in pigs. This could potentially help improve pig reproduction efficacy and advance our knowledge in human reproductive medicine.

**Abstract:**

The antral follicle stage is a critical period in mammalian oocyte maturation, marked by complex interactions between oocyte development and neighboring granulosa cells. Understanding the heterogeneity and communication patterns of granulosa cells within antral follicles is crucial for deciphering their roles in follicle development and oocyte maturation. Here, we employed single-cell RNA-sequencing to explore the molecular and cellular characteristics of porcine antral follicles. Our analysis revealed distinct subpopulations within mural and cumulus granulosa cells, indicating diverse cellular states and functions within the follicles. Functional enrichment analysis unveiled the involvement of specific subpopulations in steroid biosynthesis, cumulus expansion, and cellular communication. Moreover, comparing mature and less mature follicles highlighted differences in cell distribution and functions, indicating developmental-specific variations. Our findings shed light on the intricate cellular heterogeneity and communication network within porcine antral follicles, providing valuable insights into the regulation of follicle development and oocyte maturation in pigs. These results hold promise for improving pig reproductive efficiency and advancing human reproductive medicine.

## 1. Introduction

The antral follicle stage plays a crucial role in mammalian oocyte maturation, representing the final stages of oocyte development and ovulation. This complex process relies on synchronized interactions between oocyte maturation and the proliferation of neighboring granulosa cells [1]. During this stage, the oocyte and the surrounding somatic cells, predominantly granulosa cells, undergo critical changes and differentiation to provide essential support and hormonal regulation [2]. The regulatory system within antral follicles involves signaling from granulosa cells to the oocyte through gap junctions and the extracellular environment, facilitating oocyte meiotic arrest and restart of meiosis [3]. Consequently, comprehending the distribution of somatic cells in antral follicles is imperative for deciphering the communication signals between granulosa cells and the oocyte, as well as understanding the role of granulosa cells in oocyte development.

Recent studies have identified two subtypes of granulosa cells in antral follicles: cumulus granulosa cells (cGCs), located in the inner region, which surround and support the oocyte, and mural granulosa cells (mGCs), present in the outer layers, which provide mechanical support to the follicular wall and possess steroidogenic functions [2,4]. Single-cell RNA sequencing (scRNA-seq) investigations have unveiled granulosa cell and theca cell heterogeneity in goats, mice, and humans [5,6,7]. However, fundamental questions regarding key developmental events, granulosa cell heterogeneity, functional annotation, and the intricate somatic cell-oocyte crosstalk remain inadequately addressed at the single-cell resolution level. Moreover, communication dynamics between different types of granulosa cells, particularly in domestic animals, are still limited. Pigs, as important domestic animals and valuable models for studying human reproductive biology due to their reproductive physiology similarities, offer significant insights into follicle studies, oocyte obtainment, in vitro production, and manipulation of embryos, as well as advancements in animal husbandry and human medical progress [8].

In this study, we isolated somatic cells from porcine antral follicles and conducted scRNA-seq to analyze the single-cell transcriptomes of these cells. By identifying and sub-clustering granulosa cells, such as mGCs and cGCs, we elucidated the heterogeneity of granulosa cells in pigs. We also examined important biological processes, including hormone synthesis, cumulus expansion, and cell-cell communication. The findings from this study provide the first comprehensive characterization of granulosa cell heterogeneity in pigs, offering valuable insights into the mechanisms regulating follicle development. Moreover, these results have the potential to enhance pig reproductive efficiency, facilitate oocyte quality improvement in women with low fertility, and contribute to the advancement of both animal husbandry and human reproductive medicine. Overall, this study bridges the knowledge gap in understanding granulosa cell heterogeneity and communication in porcine antral follicles, shedding light on the molecular and cellular intricacies that govern follicular development and oocyte maturation in pigs.

## 2. Materials and Methods

### 2.1. Slaughtering Experiment

Antral follicle samples were obtained from one Landrace × Yorkshire pig that reached puberty at 210 days of age. To induce antral follicular development, the pig received an intraperitoneal injection of 5 IU of pregnant mare serum gonadotropin (PMSG) (Sigma Corporation, Guangzhou, China). After a 48-h interval, two ovaries were carefully extracted from the gilts at a local slaughterhouse and immediately transferred to the laboratory within 2 h, ensuring a temperature-controlled environment with iced 0.1% DEPC PBS to preserve sample integrity.

### 2.2. Single-Cell RNA Sequencing Library Preparation

Healthy antral follicles with a diameter exceeding 7 mm underwent a 1% Penicillin-Streptomycin PBS wash to ensure sterility. The follicles were then punctured, and the follicular fluid was gently flushed into a 60-millimeter petri dish. Cumulus-oocyte complexes (COCs) were mechanically isolated using 29 G needles, followed by careful pipetting in 0.1% hyaluronidase enzyme and subsequent washing with DPBS to remove any remaining cumulus cells. The isolated oocytes were subjected to Smart-seq2 library construction using the protocol as previously described [9]. The isolated granulosa cells suspended in the resulting fluid were centrifuged at 2000 rpm for 5 min, and the supernatant was discarded. The cell pellet was then resuspended in 100 μL of DPBS. Single-cell suspensions were processed into barcoded scRNA libraries using the 10X Genomics Chromium Next GEM Single Cell 3’ Reagent Kits v3.1, following standardized protocols. Subsequently, library construction was carried out according to the manufacturer’s instructions. The resulting libraries were subjected to pair-end sequencing with a read length of 150 bp using the Illumina HiSeq X Ten platform.

### 2.3. Quality Control

The initial processing of the raw sequencing data was carried out utilizing the CellRanger Single-Cell Software Suite (v6.0.2, 10X Genomics), encompassing alignment, filtering, and unique molecular identifier (UMI) counting. The Sscrofa11.1 (GCA_000003025.6) pig genome was employed as the reference for sequence alignment. Subsequent to cell barcode extraction, a cell-gene expression matrix was generated, which was subsequently subjected to further analysis using the Seurat R package (v3.1.0) [10]. Data filtering was performed by retaining cells with a gene count ranging from 2000 to 5000 while simultaneously ensuring that the mitochondrial content was below 7% to eliminate potentially low-quality or damaged cells. The DoubleFinder R package (v2.0.3) [11] was utilized to identify and eliminate potential doublets based on a predetermined threshold of 0.069. Following the normalization of the expression matrix, integration of the two samples obtained in this study was performed for subsequent downstream analyses.

### 2.4. Dimensionality Reduction and Cell Clustering

The top 2000 highly variable genes (HVGs) were selected for principal component analysis (PCA) to reduce data dimensionality. The 40 principal components with the highest contributions were retained for subsequent analysis. Cell clustering was performed using a graph-based algorithm. To visualize the cell clusters, we applied non-linear dimensionality reduction techniques, t-distributed stochastic neighbor embedding (t-SNE), and uniform manifold approximation and projection (UMAP). Known marker genes for different granulosa cell types were used to identify and differentiate the distinct cell types within the dataset. Mural granulosa cells and cumulus granulosa cells were further extracted, and we classified them into several subpopulations based on their gene expression profiles. Subsequent differential expression analysis was performed to identify differentially expressed genes (DEGs) among the subpopulations. Genes with an average log_e_-fold change greater than 0.5 were considered to be DEGs, and they were sorted based on adjusted *p*-values. Finally, the clusterProfiler R package (v3.14.3) [12] was used for functional enrichment analysis of differentially expressed genes, and cell subpopulations were annotated based on their functional characteristics.

### 2.5. Inference and Analysis of Cell-Cell Communication among Granulosa Cell Subpopulations

To investigate cell-cell communication networks through ligand-receptor (LR) interactions, we employed the R package CellChat (v1.1.3) [13]. The CellChat software provided a human LR database, which we adapted for use in pigs by identifying homologous genes. This allowed us to construct a pig-specific LR database. Subsequently, we created a CellChat object from a data matrix extracted from Seurat, which enabled us to model cell communication by quantifying the probabilities of communication based on the law of mass action. The communication probability represents the strength of communication between cells. We considered interactions statistically significant if the *p*-value was less than 0.05, and we filtered out communication events that were present in fewer than 20 cells.

### 2.6. Single-Cell RNA-Seq Data Analysis of Oocytes

The analysis of oocyte Smart-seq2 sequencing data in this study was conducted following the methodologies outlined in a previous published work [14]. Briefly, raw sequencing data underwent preprocessing steps, including the removal of low-quality reads and adaptors using TrimGalore (v0.6.5) (https://github.com/FelixKrueger/TrimGalore, accessed on 23 December 2022). The resulting clean reads were aligned to the reference genome (Sscrofa 11.1) using Hisat2 (v2.1.0) [15], and a read count matrix was generated utilizing featureCounts (v1.6.0) [16] with the Ensembl gene annotation (v93). Subsequently, the count matrix obtained in this study was integrated with the gene expression matrix derived from 53 oocyte samples in the aforementioned previous study. Principal component analysis (PCA) was performed on integrated data to explore the variation and relationship among the oocytes.

## 3. Results

### 3.1. Single-Cell Transcriptome Profiling Reveals Three Major Cell Types in Porcine Antral Follicles

**Figure 1:**
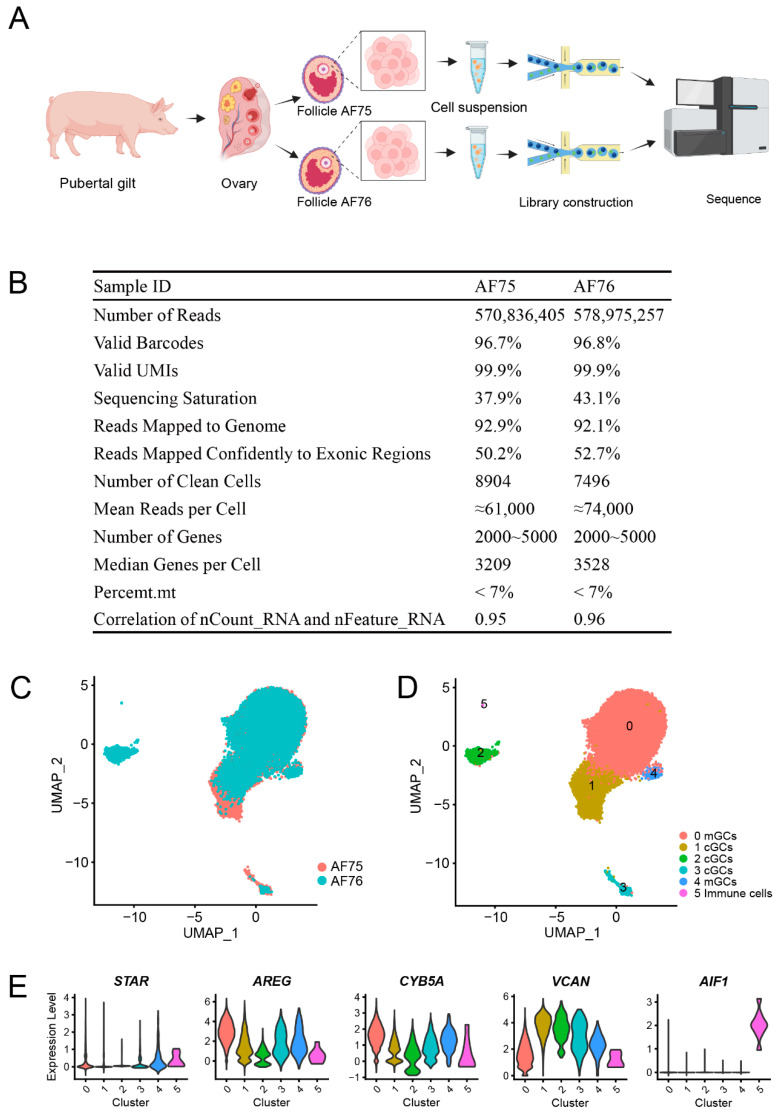
Cell clustering of somatic cells in porcine antral follicles. (**A**) Procedures for collection, preparation, and sequence of porcine antral follicular cells (created with BioRender.com). (**B**) Summary information of sample data identified by CellRanger. (**C**) Cell clusters of UMAP according to samples and (**D**) cell types. (**E**) The expression patterns of canonical marker genes in each cell cluster.

To examine the transcriptomic patterns and cell-cell crosstalk of granulosa cells in antral follicles, somatic cells of two antral follicles (each with a diameter of approximately 8.5 mm) named AF75 and AF76 were obtained and used in this investigation (Figure 1A). After having cells sequenced under the 10X Genomics scRNA-seq platform, the data were analyzed using the CellRanger program. A total of approximately 30,000 cells were captured for both AF75 and AG76, as shown in Figure 1B. The sequencing quality of both samples was assessed, with valid barcodes accounting for over 96% and valid UMIs reaching 99.9%. Additionally, more than 92% of the reads were successfully mapped to the genome, indicating the success of the sequencing process. After performing Seurat quality control and eliminating doublets, cells with gene expression levels ranging from 2000 to 5000 and mitochondrial content below 7% were retained for downstream analysis, resulting in 8904 and 7496 clean cells for AF75 and AF76, respectively. The median number of genes expressed per cell was 3209 and 3528 for the two samples, respectively. A high correlation (more than 0.95) was observed between gene count (nCount_RNA) and gene number per cell (nFeature_RNA). Utilizing the top 2000 high-variable genes from each sample, dimension reduction was performed, and subsequently, AF75 and AF76 were integrated using the top 40 principal components to identify anchors and correct for batch effects. This integration process resulted in a total of 16,400 cells (Figure 1C). It appeared that a majority of cells in both samples exhibited similar expression patterns; however, notable distinctions were observed among certain cells. Afterwards, all the integrated cells were subjected to a graph-based clustering approach using the Seurat package, resulting in the identification of six distinct clusters (Figure 1D). It is worth noting that disparities in cell distribution, particularly within clusters 1 and 2, were evident between AF75 and AF76.

We utilized canonical gene markers specific to theca cells (*LHCGR* and *STAR*) [17], mural granulosa cells (*AREG* and *CYB5A*) [18], cumulus granulosa cells (*VCAN*) [5], and other somatic cells (*AIF1*) [7,19] to characterize the cell types within the six identified clusters. Consequently, for the six cell clusters, two clusters (clusters 0 and 4) were identified as mGCs, three clusters (clusters 1, 2, and 3) as cGCs, and one cluster (cluster 5) as immune cells (Figure 1D). Notably, we did not detect expression of *LHCGR*, which is specific to theca cells. The expression heatmap for the different types of cell markers is presented in Figure 1E. Furthermore, both of the somatic cells from two antral follicles comprised three types of cells; however, there were slight differences in the cell distribution of these cell types, particularly with regard to cumulus granulosa cells.

### 3.2. Variations in Cell Distribution of Cumulus Granulosa Cells in Porcine Antral Follicles

**Figure 2:**
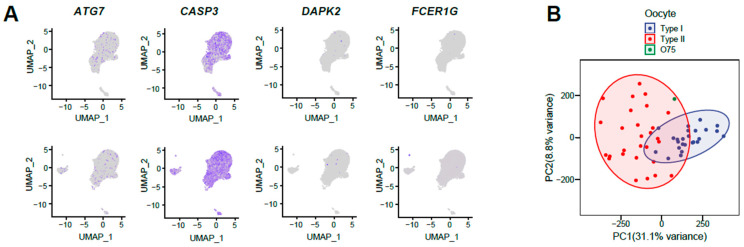
Sight differences between sample AF75 and AF76. (**A**) The UMAP plot showed the expression of genes associated with follicular atresia and degradation in AF75 (**upper**) and AF76 (**lower**), the depth of color was positively correlated with the level of expression; (**B**) PCA showed oocyte sample O75 was recognized as Type II oocytes.

In order to investigate the observed slight differences between the two follicle samples, we examined the expression of genes associated with follicular atresia and degeneration. The results presented in Figure 2A demonstrate that genes involved in cellular autophagy and apoptosis, such as *CASP3* and *ATG7* [20], did not display higher expression levels in populations of AF76. Similarly, genes associated with follicular atresia, such as *DAPK2* [21] and *FCER1G* [22], were not significantly expressed in any of the cell subpopulations.

Next, we conducted an analysis of the transcriptome features of the corresponding oocytes, which were collected from the same antral follicles as the somatic cell samples, referred to as O75 and O76. Unfortunately, only sample O75 was successfully prepared for Smart-seq2 library construction, and after sequencing and analyzing its transcriptome characteristics, we found that the gene expression profile of O75 oocytes exhibited similarities to Type II oocytes (Figure 2B), which were in a poised state for maturation [14]. As a consequence, we hypothesized that O75 was more proximal to mature oocytes, whereas the surrounding somatic cells in AF75 might represent somatic cells within the follicles that were more akin to a mature state. Based on these findings, we postulate that the greater cellular heterogeneity observed in sample AF76, as compared to AF75, can be distributed to a less mature developmental stage and a greater diversity of cell types.

### 3.3. Subpopulation Identification of mGCs and the Expression of Steroid Synthesis-Associated Genes in mGCs

**Figure 3:**
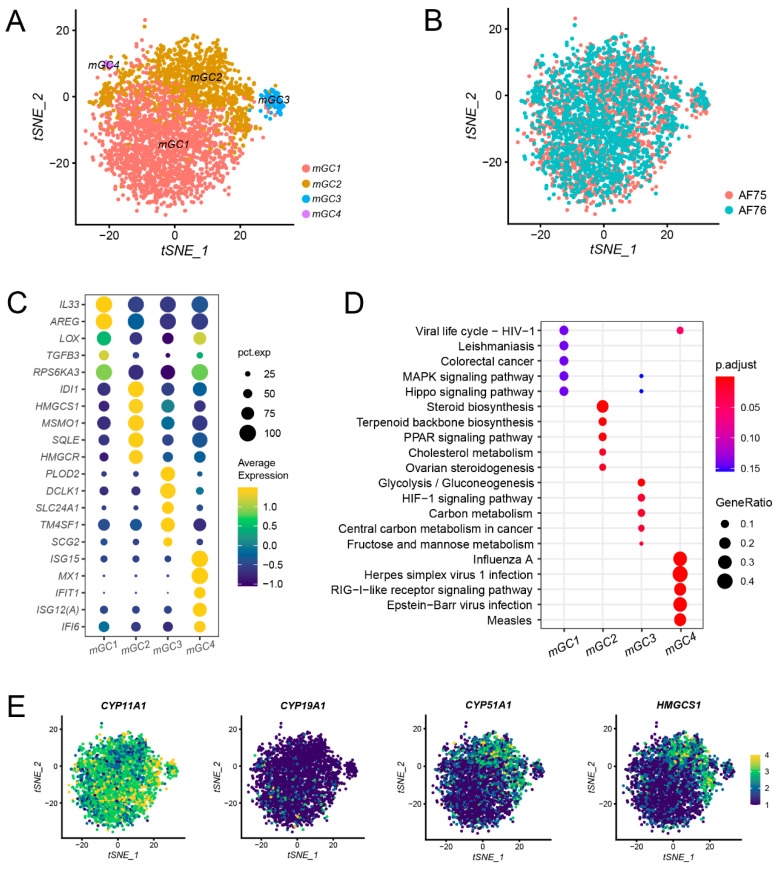
Subpopulation identification of mural granulosa cells. (**A**) The t-SNE plot showed four subpopulations of mGCs. (**B**) Cell distribution of mGCs in samples AF75 and AF76. (**C**) Five representative DEGs for each mGC subpopulation. (**D**) KEGG functional enrichment analysis of DEGs in each mGC sub-population. (**E**) Expression of genes associated with the hormone synthesis process.

To elucidate the molecular and cellular characteristics of mGCs, we isolated all mGCs and applied a graph-based clustering approach to identify subpopulations, as described previously. Subsequently, we employed the t-SNE algorithm to visualize the mGCs in a two-dimensional plot, leading to the identification of four distinct subpopulations, donated as mGC1, mGC2, mGC3, and mGC4, respectively (Figure 3A). Among these subpopulations, mGC1 and mGC2 were the predominant clusters, while mGC3 and mGC4 contained relatively small numbers of cells. The cell distribution in AF75 was notably similar to that in AF76 (Figure 3B), indicating a comparable composition and status of mGCs in the two samples.

To explore the heterogeneity of mGCs, we analyzed DEGs within each subpopulation (Figure 3C; Supplementary Table S1). Although both mGC subpopulations expressed canonical markers, their detailed transcriptomic patterns exhibited variations. For instance, DEGs in mGC1 (e.g., *AREG* and *TGFB3*) were primarily associated with signal transduction processes, while DEGs in mGC2 (e.g., *HMGCR* and *MSMO1*) were linked to cellular metabolism.

Utilizing the DEGs identified, we conducted Gene Ontology (GO) and KEGG pathway enrichment analyses to gain insights into the functional characteristics of the distinct mGC subpopulations. The enrichment results revealed specific functions associated with each subpopulation (Figure 3D; Supplementary Tables S2 and S3). For mGC1, the enriched functions included receptor ligand activity as well as MAPK and Hippo signaling pathways, indicating that cells within this subpopulation primarily engage in cellular signal processing and transduction. In contrast, mGC2 exhibited enrichment in functions related to ovarian steroidogenesis, steroid synthesis, and pathways associated with lipid metabolism. These findings suggest that cells in mGC2 play a key role in the regulation of steroid biosynthesis, which has been well-established as a crucial function of mGCs in previous studies [4,23,24]. The enriched functions in mGC3 encompassed amino acid metabolism and the cellular hormone metabolic process, suggesting that cells in this subpopulation are primarily involved in cellular metabolism. Further investigation is warranted to elucidate the specific roles of these cells in more detail. Finally, mGC4 displayed enrichment in functions related to immune responses and pathways associated with diseases, suggesting that cells within this subpopulation are mainly responsible for immune and inflammatory responses. The results of these enrichment analyses provide valuable annotations for the mGC subpopulations and offer insights into their distinct functional roles in the context of follicular development and physiology.

One of the fundamental roles of granulosa cells is to mediate hormone responses and transduction, facilitating communication between the pituitary and oocytes. Estrogen, as a pivotal ovarian hormone, plays a crucial role in promoting antral follicle growth and oocyte maturation. Here, we examined the expression of several crucial enzymes and genes associated with this hormone synthesis process. Notably, *HMGCS1* and *CYP51A1* exhibited high expression levels in mGC2, while *CYP19A1* displayed relatively low expression. On the other hand, *CYP11A1* exhibited high expression across all mGC clusters (Figure 3E). These expression patterns were consistent with the cell cluster annotation results.

### 3.4. Subpopulations Identification and Annotation of cGCs Revealed a Difference in Metabolic Capacities between AF75 and AF76

**Figure 4:**
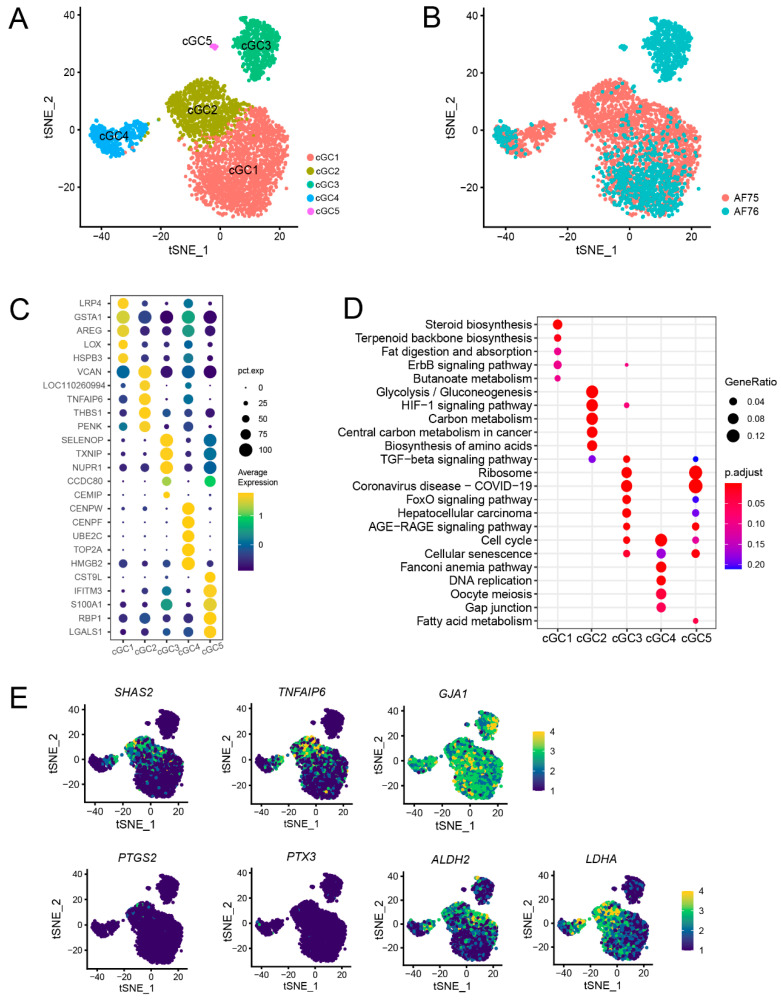
Subpopulation identification of cumulus granulosa cells. (**A**) The t-SNE plot showed five subpopulations of cGCs. (**B**) Cell distribution of cGCs in samples AF75 and AF76. (**C**) Five representative DEGs for each cGC subpopulation. (**D**) KEGG functional enrichment analysis of DEGs in each cGC sub-population. (**E**) Expression of genes associated with cumulus expansion, gap junction, and glucose metabolism.

Cumulus granulosa cells were essential for follicle development and homeostasis by providing nutrients and mechanical support for oocytes through direct contact with the zona pellucida. In this study, we isolated and clustered all cGCs into five subpopulations labeled as cGC1 to cGC5 (Figure 4A). Interestingly, sample AF76 exhibited all five subpopulations, although with relatively fewer cells of subpopulations cGC2 and cGC4 compared to AF75. Conversely, AF75 contained only three subpopulations (cGC1, cGC2, and cGC4) (Figure 4B). These findings indicate the presence of variability in the composition of cGC subpopulations between the two samples, with AF76 displaying a greater diversity of cGC subpopulations compared to AF75. Such heterogeneity in cGCs may have implications for follicular development and the regulation of oocyte maturation.

The elucidation of cellular characteristics and potential functions within the distinct subpopulations was the key to understanding the differences between samples AF75 and AF76. To achieve this, we initially attempted to annotate the cGC subpopulations by calculating DEGs for each of them. Five representative DEGs per subpopulation were then displayed (Figure 4C; Supplementary Table S4); each subpopulation exhibited unique gene expression patterns. Moreover, the results of GO and KEGG pathway enrichment analyses based on the DEGs from each subpopulation revealed significant functional differences (Figure 4D; Supplementary Tables S5 and S6). The enriched functions of cGC1 were predominantly linked to steroid biosynthesis, indicating a potential location in the outer layer of the cumulus and sharing characteristics with mGCs. In contrast, the enriched functions of cGC2 were primarily associated with glycolysis, gluconeogenesis, and amino acid biosynthesis, suggesting that this subpopulation is primarily responsible for substance synthesis and metabolism, potentially providing nutritional support to the directly interacting oocytes. Notably, cGC4 expresses genes such as *CENPF*, *UBE2C*, and *TOP2A,* which are involved in DNA replication. Correspondingly, the enriched functions of cGC4 were primarily associated with cell cycle and cell proliferation, suggesting a high proliferation index for these cells, possibly indicating their status as stem cells or progenitor cells in the follicle. Notably, the unique subpopulations cGC3 and cGC5 in sample AF76 exhibited similar functional enrichments, including pathways such as TGF-β signaling and disease processes. In contrast, AF75 corresponded to a relatively mature follicle, thus not exhibiting the heterogeneity observed in the cGC3 and cGC5 subpopulations. The number of cells of cGC1, 2, and 4 in sample AF75 was higher than AF76, indicating that AF75 had more cells with important biosynthesis and metabolism functions.

Upon LH stimulation, cGCs secrete a hyaluronic acid-rich matrix, which plays a critical role in facilitating adhesion between oocytes and cGCs. This process promotes the release of oocytes and their subsequent capture by the fallopian tube [25]. Within the cGC subpopulations, the expression of genes involved in hyaluronic acid synthesis, including *SHAS2* and *TNFAIP6*, known to be associated with cumulus expansion, was found to be significant in cGC2. Genes associated with gap junctions, such as *GJA1*, which establish connections between cumulus cells and oocytes, exhibited relatively consistent expression levels across all subpopulations. However, genes *PTGS2* and *PTX3*, previously implicated in extracellular matrix expansion [26], did not display significant expression in our samples (Figure 4E). These observations suggest that cumulus expansion relies on the concerted efforts of all cellular subpopulations, with cGC2 playing a crucial role in this process. Additionally, genes involved in glucose metabolism, such as *ALDH2* and *LDHA*, exhibited higher expression levels in subpopulations other than cGC3.

### 3.5. Inter-Subpopulation Cell-Cell Communications among Granulosa Cells

**Figure 5:**
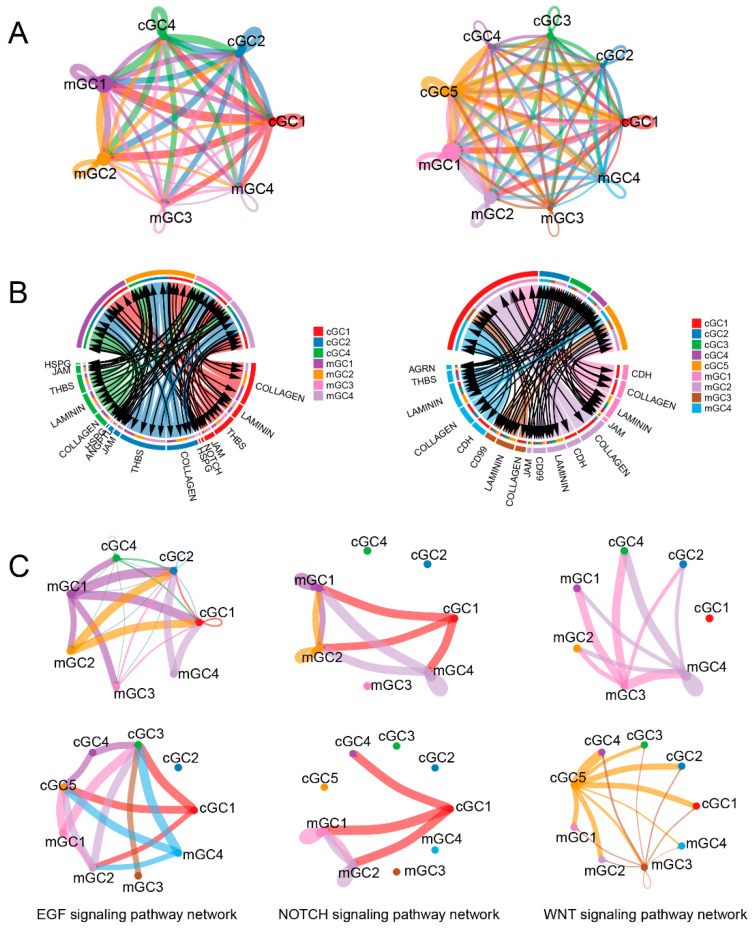
Cell-cell communications among different subpopulations of granulosa cells. (**A**) Intercommunications among various granulosa cell subpopulations in AF75 (**left**) and AF76 (**right**). (**B**) Key molecular players facilitated communications between mGC and cGC subpopulations in AF75 (**left**) and AF76 (**right**). (**C**) The transmission of the EFG, NOTCH, and WNT signaling pathways in AF75 (**upper**) and AF76 (**lower**).

We employed CellChat software to explore the communication patterns among granulosa cells within antral follicles. The results revealed extensive intercommunication among various granulosa cell subpopulations in both samples (Figure 5A). These communications encompassed signaling interactions among subpopulations of the same granulosa cell type, as well as between subpopulations of mGCs and cGCs. Notably, cGC subpopulations, such as cGC1 and cGC2, displayed higher overall activity, characterized by both widespread internal communication and the emission of numerous signaling cues to establish connections with subpopulations of mGCs. In contrast, communication among subpopulations of mGCs primarily occurred internally, with a predominant involvement of interactions between different subpopulations of the same cell type, exemplified by mGC1 and mGC2 subpopulations. These findings shed light on the intricate communication network within cumulus granulosa cells at different developmental stages, highlighting the importance of inter-subpopulation signaling interactions in coordinating follicular development and maturation processes.

The communication between mGCs and cGCs in both AF75 and AF76 samples predominantly relied on reciprocal interactions mediated by extracellular matrix (ECM) components, specifically including *COLLAGEN*, *LAMININ*, and *THBS* (Supplementary Tables S7 and S8). Furthermore, direct cell-cell contact facilitated by junctional adhesion molecules (JAMs) was found to contribute to this communication. However, the involvement of molecular interactions in establishing cellular connections appears to be limited, as molecules such as *ANGPTL*, *EGF*, *VEGF*, and *WNT* demonstrated minimal impact on this intercellular communication network. Notably, a comparison between AF76 and AF75 revealed a noticeable increase in the participation of membrane proteins CD99 and cadherin CDH in the intercommunication among cell subpopulations, signifying distinct characteristics between the two samples (Figure 5B). These findings shed light on the key molecular players that facilitate communication between mural and cumulus granulosa cells, emphasizing the significance of ECM components and cell-cell adhesion molecules in coordinating follicular development and maturation processes.

The development of ovarian follicles and the subsequent ovulation process are intricately regulated through a sophisticated interplay of signaling pathways. Among these, classical signaling pathways, such as NOTCH and WNT, assume significance in the maintenance of granulosa cell function. Previous research has demonstrated a dependence of granulosa cell proliferation on NOTCH signaling [27]. In this study, NOTCH signaling primarily flowed from cGC1 and mGC4 to mGC1 and mGC2 in sample AF75; in contrast, NOTCH signaling in sample AF76 was restricted to cGC1, targeting mGC1 and mGC2 (Figure 5C). The WNT signaling pathway, which is vital for normal folliculogenesis, luteogenesis, and steroidogenesis [28], exhibited distinct patterns between the two samples in this study. In AF75, WNT signaling mainly originated from the mGC subpopulations and influenced other subpopulations, while in AF76, the cGC subpopulations served as the principal source of WNT signaling. Furthermore, the EGF signaling pathway is an essential component of the ovulatory process as it transmits the LH signal from the periphery of the follicle to the COC [29]. Here, we observed a consistent transmission of the EGF signaling pathway from the mGC subpopulations to the cGC subpopulations in both samples. This alignment with established LH hormone-mediated transmission patterns underlines the significance of EGF signaling in this context.

## 4. Discussion

The application of scRNA-seq in farm animal investigations remains relatively limited, despite its numerous advantages. In this study, we aimed to decipher the molecular and cellular heterogeneity of granulosa cells within antral follicles and elucidate their potential roles in follicle development and oocyte maturation using 10X Genomics scRNA-seq. Our scRNA-seq analysis revealed significant heterogeneity among somatic cells within antral follicles, particularly in granulosa cells, which display distinct subpopulations with varying functions. While it has been established that somatic cells within ovarian follicles encompass two granulosa cell types [5,6], limited research has delved into the intricacies of heterogeneity within each granulosa cell type [6,7,22,30]. For the first time in pigs, our study identified distinct subpopulations within mGCs and cGCs, suggesting diverse cellular states and functions within the follicles. During the onset of puberty in mammals, theca cells in outer follicles produce androgens, which are converted into estrone and 17β-estradiol by P450 aromatase in inner mGCs, and this process is mainly orchestrated by the interaction of LH and FSH [31]. In this study, we have identified that the mGC2 subpopulation is primarily responsible for estrogen synthesis, while the mGC1 subpopulation plays a pivotal role in hormone signal transduction. The expression patterns of critical enzymes and genes associated with these processes have unveiled discernible patterns within various mGC subpopulations. Moreover, our study identifies cGC2 as responsible for glycolysis and cumulus expansion, previously considered primary functions of cGCs [6].

It is already known that in large antral follicles, a large number of follicular cells were mGCs, which contained 5~10 layers of cells, and another were cGCs, which contained 2~3 layers of cells [2]. We found the same results in our study: among 16,400 clean cells, a large amount of these were mGCs, and a small amount were cGCs. We also revealed the presence of immune cells within the follicles, consistent with findings in human and mouse ovaries [5,6,30]. The cellular interface between mGCs and cGCs likely harbors cells with characteristics of both granulosa cell types, contributing to the observed heterogeneity. Our study identified a cGC1 subpopulation that exhibits characteristics of both mGCs and cGCs. We speculate that these cells may be positioned in the outer layer of the cumulus or in close association with mGCs. It is worth noting that previous studies have also identified precursor cells shared by mGCs and cGCs, which exhibit similar traits [6]. Additionally, we identified a distinct subgroup of cGCs characterized by heightened expression of genes related to cell division and the cell cycle, potentially serving as progenitor cells, as supported by related studies [6,7]. Comparing two samples, AF75 and AF76, we noted significant distinctions in cellular composition and communication patterns. While AF75 might suggest a mature follicle, it displayed clearly delineated cGC subpopulations with specific functions, notably an increased presence of cGC2 cells responsible for cumulus expansion. Conversely, AF76 exhibited fewer cGC2 cells and distinct cGC3 and cGC5 subpopulations, hinting at a less mature follicle state. However, it is important to note that there is no definitive evidence to confirm AF75 as mature and AF76 as relatively immature at this point.

Communication within the follicle involves various mechanisms, including direct cell-to-cell contact, extracellular matrix-facilitated interactions, and molecular signaling. Extensive bidirectional signaling between granulosa cells and oocytes is crucial for proper follicle development and oocyte maturation. ErbB signaling is critical for oocyte maturation, as previously reported [32]. Recent research highlights the importance of Ras, FoxO, and insulin signaling pathways in bidirectional communication between cGCs and oocytes, with a specific emphasis on the central role of the insulin signaling pathway in regulating oocyte maturation [14]. Our study provides insights into the intricate communication network within the ovarian antral follicle, with extensive intercellular interactions mediated by gap junctions and secreted factors. Within the communication network within granulosa cells, the cGC subpopulation exhibits heightened activity. Notwithstanding these valuable discoveries, certain limitations should be acknowledged. Firstly, the limited sample size in the 10X Genomics scRNA-seq dataset makes it challenging to definitively attribute the observed distinctions between AF75 and AF76 to differences in maturational status, as opposed to inherent variations between the follicles themselves. Secondly, the Smart-seq2 library construction method employed hindered the identification of the corresponding oocyte for AF76. Consequently, accurately assessing the developmental status of follicle AF76 posed a considerable challenge. Future studies involving more oocytes and surrounding granulosa cells from follicles of varying sizes and health statuses will provide a more comprehensive understanding of granulosa cell differentiation and intercellular communication within follicles.

## 5. Conclusions

Our study focused on characterizing the molecular and cellular features of somatic cells within porcine ovarian antral follicles, leading to the identification of discrete subpopulations within both mural granulosa cells and cumulus granulosa cells. Remarkably, the intercellular communication among these subpopulations was found to be extensive, suggesting their coordinated involvement in the intricate processes of follicular development. Moreover, a comparative analysis between the two follicles revealed differences in cell distribution and function, possibly linked to varying maturation statuses. These findings significantly contribute to our comprehension of granulosa cell heterogeneity and shed light on their crucial contributions to the orchestration of follicular development.

## Data Availability

Sequence data and processed data are available under the Gene Expression Omnibus Accession GSE237134.

## Supplementary Materials

The following supporting information can be downloaded at: https://www.mdpi.com/article/10.3390/ani13193019/s1, Table S1: Differentially expressed genes (DEGs) within each mGC subpopulation. Table S2: Gene Oncology (GO) enrichment results of DEGs within each mGC subpopulation. Table S3: KEGG enrichment results of DEGs within each mGC subpopulation. Table S4: Differentially expressed genes (DEGs) within each cGC subpopulation. Table S5: Gene Oncology (GO) enrichment results of DEGs within each cGC subpopulation. Table S6: KEGG enrichment results of DEGs within each cGC subpopulation. Table S7: Inter-subpopulation cell-cell communications among granulosa cells in AF75. Table S8: Inter-subpopulation cell-cell communications among granulosa cells in AF76.
